# Heterologous coexpression of the benzoate‐para‐hydroxylase CYP53B1 with different cytochrome P450 reductases in various yeasts

**DOI:** 10.1111/1751-7915.13321

**Published:** 2018-10-19

**Authors:** Chrispian W. Theron, Michel Labuschagné, Jacobus Albertyn, Martha S. Smit

**Affiliations:** ^1^ Department of Microbial, Biochemical and Food Biotechnology University of the Free State Bloemfontein South Africa; ^2^ South African DST‐NRF Centre of Excellence in Catalysis, c*change University of Cape Town Cape Town South Africa

## Abstract

Cytochrome P450 monooxygenases (P450) are enzymes with high potential as biocatalysts for industrial applications. Their large‐scale applications are, however, limited by instability and requirement for coproteins and/or expensive cofactors. These problems are largely overcome when whole cells are used as biocatalysts. We previously screened various yeast species heterologously expressing self‐sufficient P450s for their potential as whole‐cell biocatalysts. Most P450s are, however, not self‐sufficient and consist of two or three protein component systems. Therefore, in the present study, we screened different yeast species for coexpression of P450 and P450‐reductase (CPR) partners, using CYP53B1 from *Rhodotorula minuta* as an exemplary P450. The abilities of three different coexpressed CPR partners to support P450 activity were investigated, two from basidiomycetous origin and one from an ascomycete. The various P450‐CPR combinations were cloned into strains of *Saccharomyces cerevisiae, Kluyveromyces marxianus, Hansenula polymorpha, Yarrowia lipolytica* and *Arxula adeninivorans*, using a broad‐range yeast expression vector. The results obtained supported the previous finding that recombinant *A. adeninivorans* strains perform excellently as whole‐cell biocatalysts. This study also demonstrated for the first time the P450 reductase activity of the CPRs from *R. minuta* and *U. maydis*. A very interesting observation was the variation in the supportive activity provided by the different reductase partners tested and demonstrated better P450 activity enhancement by a heterologous CPR compared to its natural partner CPR. This study highlights reductase selection as a critical variable for consideration in the pursuit of optimal P450‐based catalytic systems. The usefulness of *A. adeninivorans* as both a host for recombinant P450s and whole‐cell biocatalyst was emphasized, supporting earlier findings.

## Background

The cytochrome P450 monooxygenases (P450s) are a promising group of enzymes for industrial applications, as they are capable of catalysing the stereo‐ and regiospecific hydroxylation of non‐activated C–H bonds. As such, they are of interest in diverse research fields, most notably research on drug metabolism and design, fine chemical synthesis and bioremediation (Kumar, 2010; O'Reilly *et al*., 2011; Schewe *et al*., 2011). Large‐scale applications of these enzymes have, however, been limited by their requirement for expensive cofactors, and in most cases, coproteins are also required for catalysis to occur, while poor enzyme stability is a further limitation (Gillam, 2007; O'Reilly *et al*., 2011; Urlacher and Girhard, 2012). These limiting factors can be greatly circumvented by employing whole‐cell systems for bioconversions, necessitating the identification of appropriate hosts for heterologous expression of P450s (Lundemo and Woodley, 2015).

Although *Escherichia coli* is generally the universal first‐choice host for heterologous expression, it is limited in P450 production, particularly when eukaryotic P450s are targeted (Waegeman and Soetaert, 2011; Urlacher and Girhard, 2012). While bacterial P450s tend to have higher activities and improved stability over eukaryotic P450s, the latter are of interest due to interest in their roles in drug design and metabolism, making them important tools in human medical research (Kumar, 2010) as well as potential targets for antifungal drugs (Kelly and Kelly, 2013; Jawallapersand *et al*., 2014). Furthermore, current nucleic acid sequencing technologies are revealing vast numbers of P450s in plants (Mizutani, 2012) and even more so in fungi (Floudas *et al*., 2012; Ichinose and Wariishi, 2013), representing immense reservoirs of potentially useful enzymes requiring functional characterization. Fungi in particular harbour a multitude of catalytically diverse P450s yet to be completely explored and harnessed (Durairaj *et al*., 2016).

Yeasts are attractive alternative hosts for expression of eukaryotic P450s, since they combine eukaryotic protein machinery with prokaryotic simplicity of growth and manipulation. In a previous study, we used a broad‐range vector system developed in our research group (Smit *et al*., 2012) to compare the heterologous expression of self‐sufficient P450s by various yeast species simultaneously and using consistent biotransformation procedures (Theron *et al*., 2014). The majority of P450s are, however, not self‐sufficient, single‐component systems, but tend to rely on one or two additional protein components for cofactor supply. Therefore, to broaden the applications of the aforementioned vector system, we investigated the expression of a non‐self‐sufficient P450.

CYP53B1, a benzoate‐*para*‐hydroxylase (BpH) from *Rhodotorula minuta* (recently reclassified as *Cytobasidium minutum*), was chosen as an exemplary fungal P450, as CYP53 family members have been observed across all subclasses of basidiomycetous fungi, as well in the Pezizomycotina subclass of *Ascomycetes* (Jawallapersand *et al*., 2014). The hydroxylation of benzoic acid (BA) and its derivatives is a critical step in degradation of structural and protective compounds of plants, and as such these enzymes have been linked to persistence and pathogenesis of fungi to plants (Jawallapersand *et al*., 2014; Durairaj *et al*., 2016). In fact, this is one of three P450 families highly conserved among biotrophic plant pathogenic fungi, with the other two families involved in membrane ergosterol biosynthesis, highlighting their importance (Qhanya *et al*., 2015). As such, they have been identified as potential targets for antifungal drugs (Jawallapersand *et al*., 2014; Korošec *et al*., 2014). BA and the CYP53B1 hydroxylation product, *p*‐hydroxybenzoic acid (pHBA), are industrially versatile chemicals, with applications as intermediates in drug synthesis, in food preservatives and polyester fibres (Durairaj *et al*., 2015b). In addition, we previously established reasonably rapid and convenient assays for detection and quantification of BA and pHBA (Shiningavamwe *et al*., 2006; Theron, 2007).

Recombinant P450 activities are usually improved by the coexpression or supplementation of the natural reductase partners (Gudiminchi *et al*., 2013; Syed *et al*., 2013), or alternatively by overexpressing the native reductase of the host (Nthangeni *et al*., 2004; Schiffler *et al*., 2004). Exploration of the different effects of non‐related (heterologous) reductases on P450 activity is, however, scarce. To support CYP53B1 activity in this study, we coexpressed it in combination with three different CPRs: the natural CPR partner from *R. minuta* (RmCPR); the CPR from another basidiomycete *Ustilago maydis* (UmCPR), which is related to *Rhodotorula* spp. (Teichmann *et al*., 2007); and the ascomycetous *Y. lipolytica* CPR (YlCPR), which was previously used in our research group (Shiningavamwe *et al*., 2006). The ascomycetous CPR was chosen due to the ascomycetous nature of yeast hosts investigated in this study: *Saccharomyces cerevisiae*,* Yarrowia lipolytica*,* Arxula (Blastobotrys) adeninivorans, Hansenula (Ogataea) polymorpha* and *Kluyveromyces marxianus*.

## Results

### Cloning of basidiomycetous CPRs

Total RNA was extracted from *R. minuta* CBS2177 and reverse‐transcribed to cDNA, which served as a template for the specific PCR amplification of the RmCPR, based on the deposited sequence (GenBank:AB055119). The deposited sequence was annotated as a P450 reductase, although to our knowledge activity verification has not been performed prior to the current study. The cloned gene was verified by sequence analysis and had only one nucleotide difference with the deposited sequence. The intronless *U. maydis* CPR (UmCPR) ORF was PCR amplified directly from genomic DNA extracted from the DSM3121 strain, using primers based on the deposited sequence annotated as a putative NADPH‐cytochrome P450 reductase. The cloned gene was again verified by sequence analysis. It showed six nucleotide differences when compared with the deposited sequence from *U. maydis* strain 521 with five of these silent mutations. Both these new CPR‐encoding sequences were submitted to GenBank (RmCPR – KY586135; UmCPR – KY586136).

### Manipulation of the broad‐range expression vector to obtain constructs for coexpression

We had previously developed a vector system that allows expression in a broad range of yeast hosts, based on components that are either conserved between yeast species, or that were shown to be functional in a range of yeasts. (Smit *et al*., 2012; Theron *et al*., 2014). Two versions of the vector, containing one and two I‐SceI restriction sites, respectively, allow construction of a coexpression vector *via* selective recombination events.

**Figure 1:**
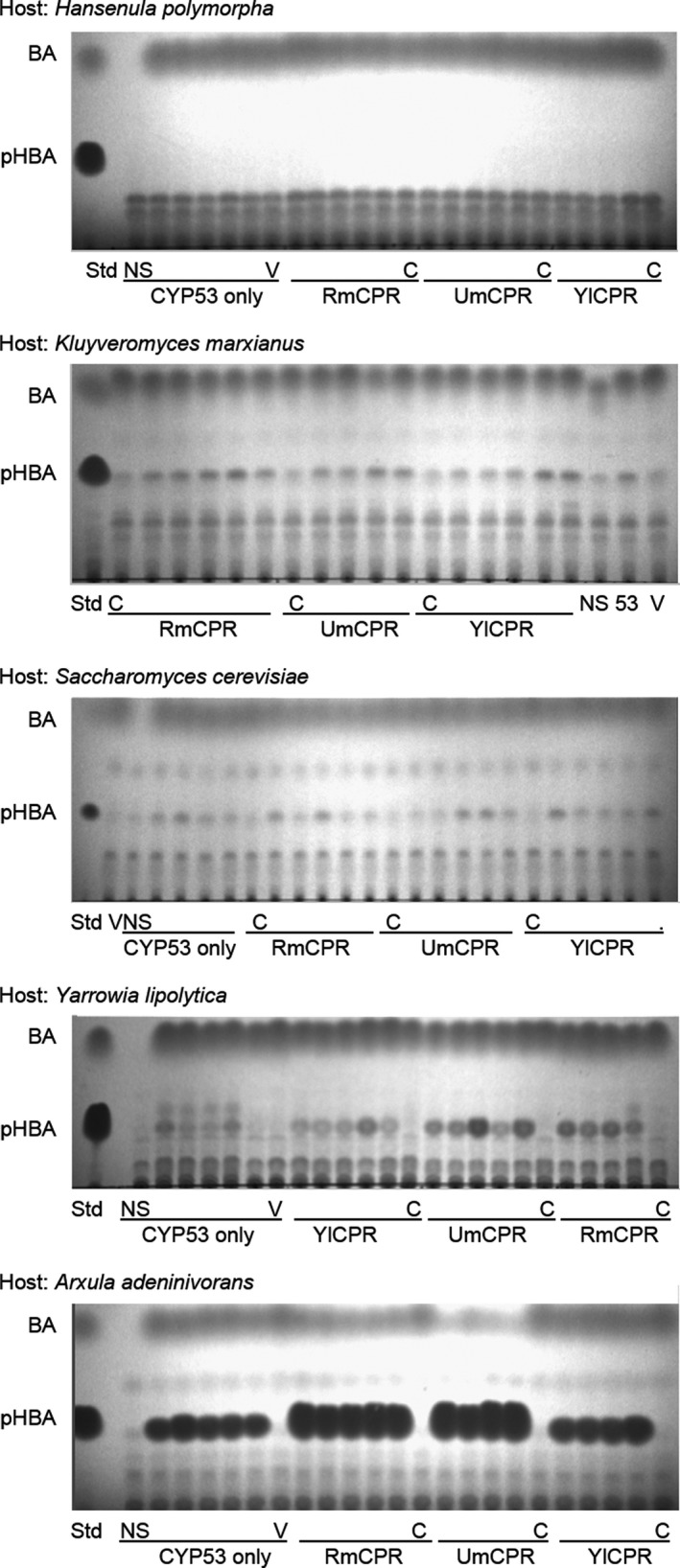
TLC analysis of the initial screening of BpH activities of transformants of *H. polymorpha*,* K. marxianus*,* S. cerevisiae*,* Y. lipolytica* and *A. adeninivorans*. Std – benzoic acid (BA) and *p‐*hydroxybenzoic acid (pHBA) standards. NS – CYP53B1‐expressing transformant to which no substrate was added. V – transformant containing pKM173 without CYP53B1 or any CPR. RmCPR – CPR from *R. minuta*, UmCPR – CPR from *U. maydis*, YlCPR – CPR from *Y. lipolytica*. C – transformant containing only CPR without CYP53B1.

For this purpose, the amplified reductase genes YlCPR, RmCPR and UmCPR were separately cloned into the broad‐range vector pKM173, which contains only one I‐SceI restriction site immediately preceding the YlTEF promoter (i.e. preceding the expression cassette). The amplified CYP53B1 gene was cloned into pKM177, which has I‐SceI restriction sites on both sides of the expression cassette. The expression cassette was thereafter removed from pKM177 by digestion with I‐SceI and recombined by ligation with the three different pKM173 vectors, each containing a different CPR, which had been opened with the same enzyme. This resulted in vectors for coexpression of each CPR with CYP53B1, for integration into various yeast hosts for comparative activity analysis (Fig. 1).

### Initial screening of transformants expressing CYP53B1

After confirmation of genomic integration of relevant genes of interest by PCR, 4–5 transformants from each parental strain containing CYP53B1, with or without co‐integrated foreign CPRs, were screened for whole‐cell benzoate‐*para*‐hydroxylase (BpH) activities. These were compared to various control reactions to account for any potential non‐specific interfering background compounds. It was demonstrated that all of the tested yeasts reached stationary phase in YPD medium within 48 h (Theron, 2012), and therefore, benzoic acid (BA) dissolved in DMSO was added directly to the culture medium after 48 h cultivation. Samples were taken regularly during further incubation on the rotary shaker. Benzoate‐*para*‐hydroxylase (BpH) activity was monitored using thin‐layer chromatography (TLC) analysis. The TLC results for the 24 h samples are shown in Fig. 1, with samples taken at later times not showing significantly improved results.

The tested *H. polymorpha* strain was the only host for which no activity could be detected. The most promising results obtained during the screening were with transformants of *A. adeninivorans*, which produced more *p*‐hydroxybenzoic acid (pHBA) than transformants of the other yeasts. The effect of the different CPRs on BpH activities was therefore also the most pronounced in transformants of this yeast. The most promising CYP53B1‐containing transformants identified during the initial screening were selected for further biotransformation experiments. In addition, since the combination of UmCPR with CYP53B1 yielded favourable results in all of the hosts tested, the most promising transformants containing this combination were also selected for further biotransformation experiments.

**Figure 2:**
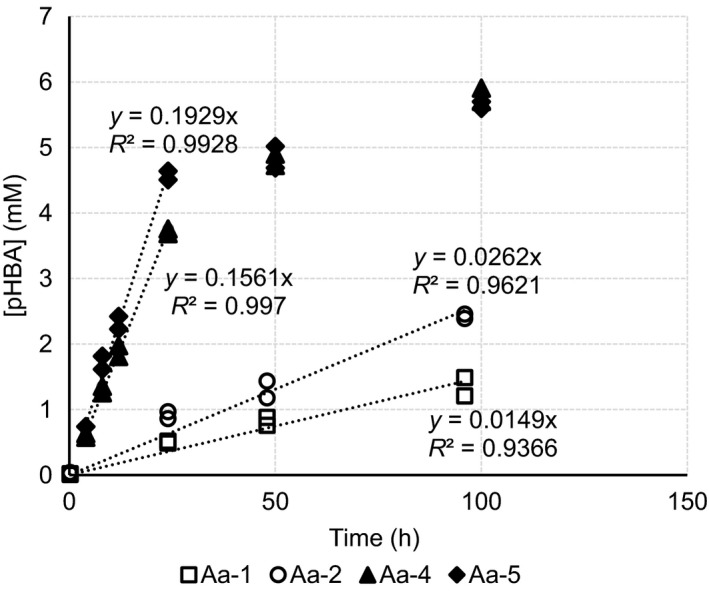
Progress curves of CYP53B1 activity in *A. adeninivorans* transformants. Aa‐1 and Aa‐2 expressing only wild‐type CPR. Aa‐4 and Aa‐5 coexpressing UmCPR. Values are for two independent reactions carried out with 48 h cultures of two different transformants.

The highest activities observed were with *A. adeninivorans* coexpressing CYP53B1 and UmCPR. Progress curves were therefore constructed using data accumulated using these transformants as well as transformants expressing only CYP53B1. Biotransformations were performed by adding the substrate directly to the 48 h cultures. The formation of the pHBA was quantified using gas chromatography (GC) analysis. These results demonstrated that with the transformants coexpressing CYP53B1 and UmCPR, the reaction rates were still linear by 24 h of biotransformation, reaching a plateau later on (Fig. 2). Reliable data for samples earlier than 24 h were scarce for most other yeasts tested, due to far slower reaction rates. The 24 h time point was therefore selected for comparative activity determinations, as the other reaction rates were deduced to also still be in the linear phases at this point. The reliability of this approach was demonstrated by comparing activities calculated using the 24 h samples to the gradients of the linear phases of the progress curves (up to 24 h) for *A. adeninivorans* transformants (Table S3).

### Biotransformations using transformants of various yeast species expressing CYP53B1 only

**Figure 3:**
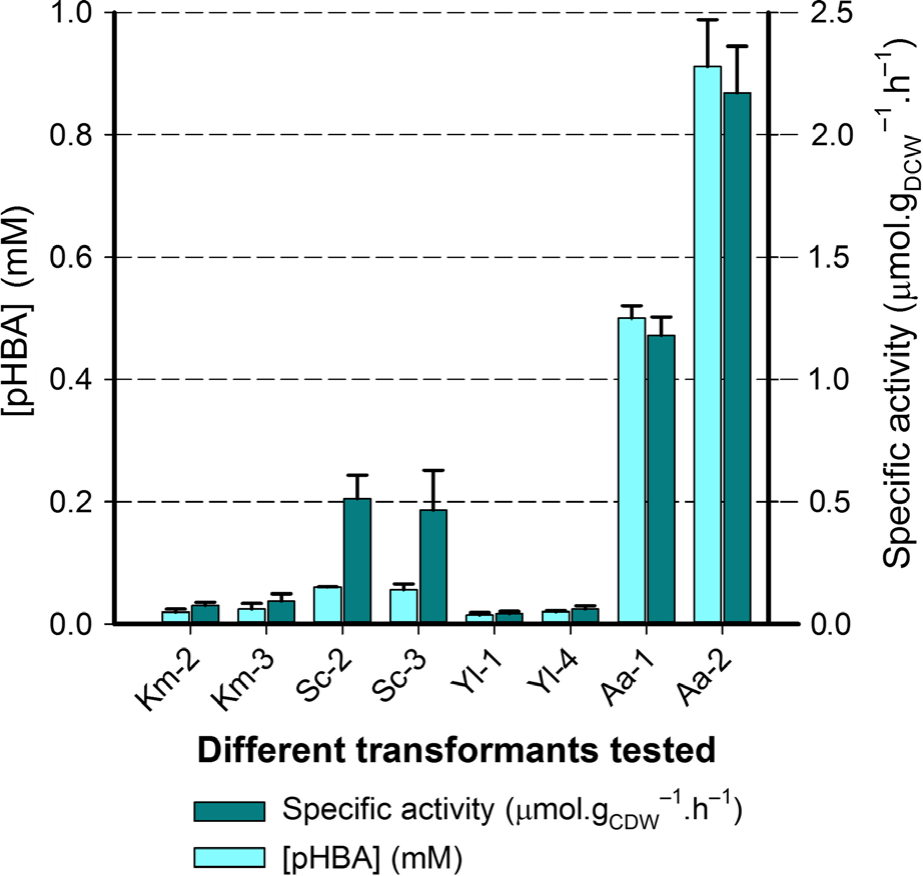
pHBA production and specific activity for 24 h reactions carried out with transformants of *K. marxianus* (Km), *S. cerevisiae* (Sc), *Y. lipolytica* (Yl) and *A. adeninivorans* (Aa) heterologously expressing CYP53B1 alone. Values are for two independent reactions carried out with 48 h cultures of two different transformants (Table S4).

The best performing transformants from the initial screening of each species were selected for direct interspecies comparisons. Activities were considered in terms of volumetric product yield as well as specific activities calculated based on the biomasses of 48 h samples (Fig. 3). Since biomass yields would naturally vary between different yeasts under the applied conditions, specific activities determined by normalization with biomasses improved comparison between strains. Specific activities were determined using biomasses produced by these strains after 48 h growth (i.e. at the time of substrate addition), which varied significantly between the yeasts (Table S2), but not much between different transformants. Biomasses did not significantly change after addition of BA.

The best results were obtained with *A. adeninivorans* transformants, with a maximum volumetric yield of 0.91 mM of pHBA formed at a specific rate of 2.17 μmol.h^−1^ g_DCW_^−1^. The other yeasts ranked in terms of performance (from next highest to lowest activities): *S. cerevisiae*,* K. marxianus* and *Y. lipolytica*. Toxicity of BA or pHBA due to low pH of cultures of the *S. cerevisiae* and *K. marxianus* transformants (Table S2) was ruled out as a cause for lower activities, as the use of buffered YPD media which maintained the pH between 7 and 8 did not improve activities of these transformants (Theron, 2012). There appeared to be a twofold difference in activity between the two transformants of *A. adeninivorans* tested in this experiment (Fig. 3). The doubled activity could possibly indicate a copy number difference between these transformants, although this was not confirmed. Nevertheless, the *A. adeninivorans* transformant with lower activity still produced more than eightfold more pHBA than the next best producer, *S. cerevisiae* transformant 2 within 24 h, and at more than double the specific rate.

### Analysis of the differential effects of cytochrome P450 reductases (CPR) on CYP53B1 activity

**Figure 4:**
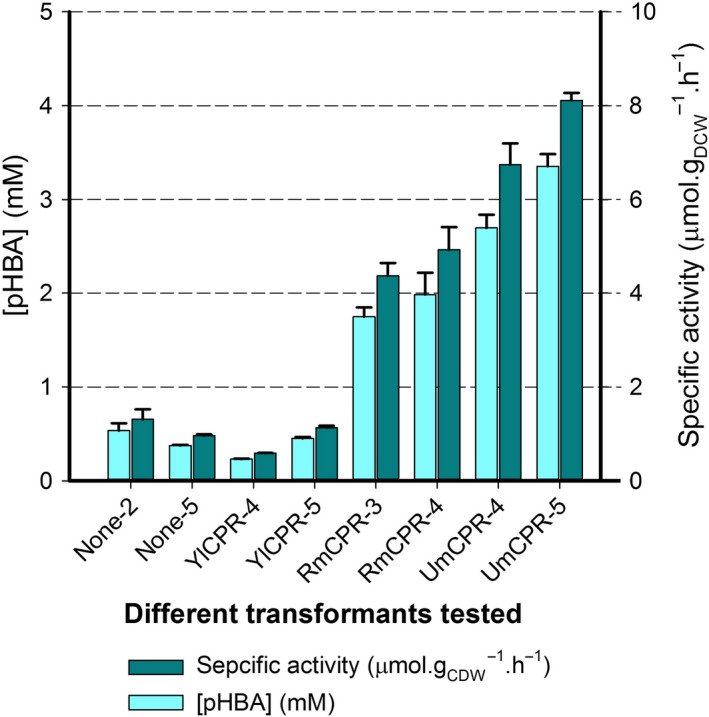
pHBA production and specific activity for 24 h reactions carried out with transformants of *A. adeninivorans* heterologously expressing CYP53B1 alone (none) or coexpressing CYP53B1 with the CPRs from *Y. lipolytica* (YlCPR), *R. minuta* (RmCPR) or *U. myadis* (UmCPR). Values are for two independent reactions carried out with 48 h cultures of two different transformants (Table S5).

Since the most promising results obtained during the screening were with transformants of *A. adeninivorans*, the effects of the different CPRs on BpH activities were the most pronounced in these transformants (Fig. 1). A separate set of biotransformation experiments was therefore performed to directly compare *A. adeninivorans* transformants coexpressing foreign CPRs with CYP53B1, to strains heterologously expressing only CYP53B1 (Fig. 4; Table S5).

Coexpression of the CPR from *U. maydis* (UmCPR) resulted in the greatest increase in activity, with sixfold to sevenfold increases in 24 h volumetric yield (2.7–3.35 mM) of pHBA compared to when only the native CPR was available (0.37–0.54 mM). The specific activity also increased by sixfold to sevenfold from 0.96 to 1.31 μmol.h^−1^ g_DCW_^−1^ with only the native CPR compared to 6.74–8.11 μmol.h^−1^ g_DCW_^−1^ when UmCPR was coexpressed. As expected, the CPR from *R. minuta* (RmCPR) also enhanced the activity, with volumetric yields of 1.75–1.99 mM of pHBA (roughly fourfold increases) and specific activities of 4.37–4.93 μmol.h^−1^ g_DCW_^‐1^ (also roughly fourfold increases). The CPR from *Y. lipolytica* (YlCPR) on the other hand did not enhance the activity of CYP53B1 compared to the native CPR.

### Enhancement of BpH activity in other yeast hosts by coexpression of UmCPR with CYP53B1

**Figure 5:**
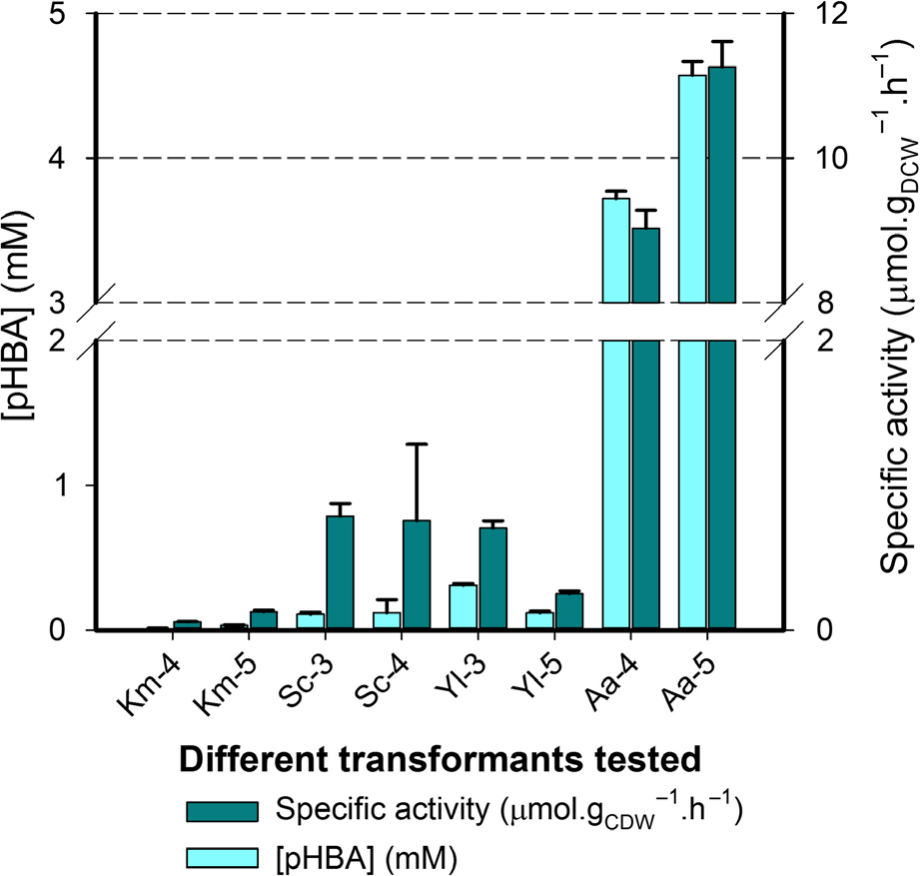
pHBA production and specific activity for 24 h reactions carried out with transformants of *K. marxianus* (Km), *S. cerevisiae* (Sc), *Y. lipolytica* (Yl) and *A. adeninivorans* (Aa) coexpressing CYP53B1 with the CPR from *U. maydis*. Values are for two independent reactions carried out with 48 h cultures of two different transformants (Table S4).

Since the combination of UmCPR with CYP53B1 appeared to yield equal or improved conversions of BA in all host strains, the best performing transformants coexpressing UmCPR with CYP53B1 for each strain were selected for comparison with their CYP53B1 only counterparts. This comparison would determine the effects of coexpression of this reductase on recombinant BpH activity in the different strains tested (Fig. 5; Table S4). As before, 24 h samples were used for activity analysis.

*K. marxianus* transformant 5 (T5) had nearly double the activity of transformant 4 (T4), while *Y. lipolytica* transformant 3 (T3) had nearly triple the activity of transformant 5 (T5), again possibly indicating differences in copy numbers between these transformants, although this was not confirmed. These differences were, however, kept in mind during result interpretation.

The coexpression of UmCPR proved to have variable effects among the species, with far higher BpH activity improvements in *Y. lipolytica* and *A. adeninivorans* than in *K. marxianus* and *S. cerevisiae*. The improvement in activity caused by the coexpression of UmCPR was comparable in *Y. lipolytica* and *A. adeninivorans*. In *Y. lipolytica*, the volumetric yields increased from the previous highest yield of 20 μM to 119 μM and 308 μM of pHBA for transformants 5 and 3 respectively. Meanwhile, the previous highest specific activity of 0.061 μmol.h^−1^ g_DCW_^−1^ was increased to 0.25 μmol.h^−1^ g_DCW_^−1^ for transformant 5 and 0.7 μmol.h^−1^ g_DCW_^−1^ for transformant 3. *A. adeninivorans,* however, still showed the highest overall activity, with bioconversion yields of 74% (T4) and 91% (T5). The obtained reaction rates for selected transformants from this yeast were on average sixfold higher with the coexpression of UmCPR. Product yields in *S. cerevisiae* doubled with the coexpression of UmCPR, while recombinant BpH activity in *K. marxianus* was hardly affected by the inclusion of this CPR.

## Discussion

Whole‐cell biocatalysis is an attractive option to alleviate problems often encountered with cytochrome P450‐based applications, such as enzyme instability and the supply and regeneration of cofactors (Lundemo and Woodley, 2015). Suitable hosts are, however, required for recombinant enzyme production, particularly eukaryotic hosts for eukaryotic P450s. For instance, multitudes of uncharacterized fungal P450s are being uncovered, potentially representing a diverse wealth of catalysts (Floudas *et al*., 2012).

In a previous study, we demonstrated the superior performance of *A. adeninivorans* during the screening of various yeasts for the production of catalytically self‐sufficient, single‐component P450 systems. This was done through the use of a broad‐range expression vector developed in our group that is based on components that are either conserved between yeast species or that were shown to be functional in a range of yeasts (Theron *et al*., 2014). The majority of P450 systems are, however, multicomponent in nature, leading us to test the applicability of our expression system towards P450‐CPR combinations in this study. Two versions of the broad‐range expression vector, differing only in the number of I‐SceI restriction sites they contain, allow construction of a coexpression vector via selective recombination events. This option is ideally suited for P450‐based applications where an essential reductase partner can be coexpressed with the P450 in tandem expression cassettes.

We selected CYP53B1 as a representative fungal P450. CYP53 is an important P450 family in biotrophic plant pathogens and has been investigated as an antifungal drug target (Berne *et al*., 2012; Korošec *et al*., 2014). *p*‐Hydroxybenzoic acid (pHBA), the product of CYP53B1 from BA, is a versatile intermediate compound during chemical synthesis. Furthermore, it is a useful exemplary P450 for expression using ascomycetous yeast, due to its natural absence in the genomes of this fungal class (Kgosiemang *et al*., 2014), thus excluding interference by natural background activities. The absence of these enzymes from ascomycetes compared to fungal genomes is not surprising given the drastic lifestyle differences between these fungal classes, as well as the smaller genome sizes and generally lower P450 content.

The vector system proved to be efficient for expression of CYP53B1, as well as the coexpression of different CPR partners towards catalysis in different yeast strains. CYP53B1 was co‐integrated with each CPR as tandem expression cassettes in the same integration cassette, ensuring an equal P450:CPR ratio in all cases. We demonstrated for the first time to our knowledge, the P450 reductase activity of the RmCPR and UmCPR. Probably, the most interesting observation of this study was the considerable variation in effects of the different CPRs tested on CYP53B1 activity. Naturally, differences were expected, since the basidiomycetous UmCPR and RmCPR share 55% amino acid identity, while they share only 43% and 44% amino acid identity, respectively, with the ascomycetous YlCPR. It is furthermore not surprising that the basidiomycetous UmCPR and RmCPR enhanced the activity of the CYP53B1 (also from basidiomycetous origin) better than the YlCPR, despite the hosts all being ascomycetous yeasts. This suggested a probable higher compatibility between the basidiomycetous CPRs and P450. The more surprising finding was that UmCPR from *U. maydis* (subphylum: Ustilaginomycotina) had a more prominent effect on CYP53B1 than RmCPR from *R. minuta* (subphylum: Pucciniomycotina), the natural CPR partner of CYP53B1. Historically, studies involving heterologous P450 expression have generally compensated for insufficient electron supply from natural CPR systems by either overexpressing the native CPR of the host (Nthangeni *et al*., 2004; Schiffler *et al*., 2004) or coexpressing the natural CPR partner from the P450 source organism (Gudiminchi *et al*., 2013; Syed *et al*., 2013). Both of these approaches were recently applied in studies on the expression of benzoate hydroxylases and fatty acid ω‐hydroxylases from *Fusarium oxysporum* in *Saccharomyces* cerevisiae (Durairaj *et al*., 2015a,b). Regardless of the *F. oxysporum* P450 expressed in *S. cerevisiae*, the cooverexpressed *F. oxysporum* CPR (source) enhanced activity better than the overexpressed *S. cerevisiae* CPR (host). Additionally, when the unrelated CPR from *Candida albicans* was also overexpressed with CYP53A19 in *S. cerevisiae*, poorer results were obtained than in both of the other cases (Durairaj *et al*., 2015b). Interestingly, CPR assays performed in that study on CPRs in isolation indicated relatively similar activities among all of the CPRs tested, suggesting that differences in P450 activities were due to specific CPR‐P450 interactions. Furthermore, as the CYP53A19 activity increased, so did the range of reactions catalysed, indicating that unfavourable P450‐CPR interactions support primarily essential functions, while favourable interactions allow additional functions. This may, however, also be explained by undetectably low levels of additional products formed due to lower overall activities.

Further demonstration of the complicated nature of P450‐CPR interactions was obtained in a fascinating study by Lah *et al*. (2011). Many fungal species contain only one CPR regardless of the number of P450s present (Lah *et al*., 2008), including ascomycetous Pezizomycotina and basidiomycetous Agaricomycotina, which can encode hundreds of potential P450s in their genomes (Lah *et al*., 2008, 2011; Qhanya *et al*., 2015). One CPR is therefore capable of supporting numerous P450s, although in certain organisms more CPRs are present. The study by Lah and coworkers (Lah *et al*., 2011) included one such case, in which the interaction of the two native CPRs with the CYP53A15 from *Cochliobolus lunatus* was investigated. They demonstrated that not only does the constitutively expressed CPR1 react more favourably with CYP53A15 than the CPR2 (which is induced by derivatives of benzoate), but that the product profiles also differed. When reconstituted with CPR1, CYP53A15 converted BA to pHBA and *m*‐methoxybenzoate to *m*‐hydroxybenzoic acid (mHBA), while both substrates were converted to 3,4‐dihydroxybenzoic acid when reconstituted with CPR2. They concluded that CPR1 plays an important role in primary metabolism, while CPR2 is involved in secondary metabolism, including xenobiotic compound degradation. One could also consider it as a detoxification versus a carbon‐scavenging role, since 3,4‐dihydroxybenzoic acid is primed for further breakdown potentially as alternative carbon source in the absence of preferred carbon sources. Either way, it is both clear and remarkable that different physical interactions between CPRs with the same P450 can alter not only the conversion rate but also the product profile of the P450. This is likely the explanation for our findings as well that the preferential physical interaction between UmCPR and CYP53B1 on the molecular level facilitates improved activities over the RmCPR‐CYP53B1 combination.

The fact that YlCPR did not improve CYP53B1 activity compared to *A. adeninivorans* native CPR supports this theory. This is significant because *Y. lipolytica* has exceptionally high P450 content among ascomycetous yeasts, with 17 P450s (Nelson, 2009). Twelve of these belong to the CYP52 family and are involved in alkane and fatty acid metabolism, allowing this organism to efficiently degrade these types of compounds (Fickers *et al*., 2005; Fukuda and Ohta, 2013). Therefore, the CPR is clearly efficient by nature, but probably not ideally compatible with the CYP53 family that is absent from the Saccharomycotina subphylum. This is further emphasized by the fact that YlCPR coexpression with CYP53B1 in *Y. lipolytica* itself did not yield better activities than the UmCPR. In such cases, the heterologous expression of a non‐efficient partner probably caused unproductive metabolic burden on the strains. The higher cooperativity of UmCPR with CYP53B1 than RmCPR may be attributable to the higher reliance on CYP53 by *U. maydis* due to its previously discussed proposed role in plant pathogenesis and neutralization of plant defence compounds, which would require highly efficient interactions for high reaction rates. By contrast, the saprophytic lifestyle of *R. minuta* is likely to be far less reliant on CYP53.

Considering the general catalytic cycle of P450‐mediated reactions, substrate binding precedes haem reduction by reductase proteins (Isin and Guengerich, 2008). This is stated to be at least partially due to a change in the spin state of the catalytic iron, increasing the thermodynamic feasibility of reduction. This can also be considered a protective mechanism to prevent the reduction in the absence of substrate that could cause generation of harmful reactive oxygen species. Furthermore, structural evidence obtained using nuclear magnetic resonance (NMR) studies, among other techniques, indicates conformational shifts in the P450 structure upon substrate binding (Colthart *et al*., 2016). Naturally, positional changes of residues in the substrate access predominate, however residues far from the active site are also affected. It has been suggested that such conformational changes affect interaction of a P450 with a reductase partner (Zhang *et al*., 2014). It could therefore be argued that conformational changes brought about by binding of an ideal substrate facilitate optimal interaction between the reductase and P450, due to the correct positioning and distance between the FMN of the CPR and the haem group of the P450. If this is the case, then binding of other substrates would facilitate less efficient interaction, as appears to be evidenced by studies linking spin state of catalytic iron to NADPH consumption (Colthart *et al*., 2016).

It can be speculated that the interaction of P450s with foreign CPRs follows a similar tendency. It is plausible that CPRs in species like yeasts with comparatively few P450s evolve to facilitate ‘familiar’ reactions, based on the predominating P450s present. It was argued earlier that *U. maydis* is likely to rely on CYP53 during plant pathogenesis; therefore, it makes sense that its CPR is highly capable of interacting efficiently with these enzymes. Likewise for *R. minuta*, although this yeast possibly relies on CYP53 to a lesser extent. Since the CYP52 family dominates in *Y. lipolytica* however, the fatty acid/alkane substrates that bind are likely to induce considerably different conformational changes than benzoate derivatives. Taken together with the lack of CYP53 family members in *Y. lipolytica*, this is a possible explanation for facilitation of more efficient P450 reactions.

Considering overall CYP53B1 activities, in accordance with previous work, *A. adeninivorans* was found to be the most promising host, with CYP53B1 activities far surpassing the other yeasts tested with or without coexpressed CPRs. While the coexpression of UmCPR had a generally positive effect on BpH activity in all tested hosts, the highest activity of a transformant from another host containing the UmCPR‐CYP53B1 combination, that is *S. cerevisiae* T4, could not reach the level of the lowest *A. adeninivorans* transformant T1 expressing only CYP53B1, that is supported by the native reductase systems. *A. adeninivorans* has previously exhibited favourable heterologous protein production for various proteins compared to other yeasts (examples include Böer *et al*., 2007; Smit *et al*., 2012; Theron *et al*., 2014; Kumari *et al*., 2015; Kasprzak *et al*., 2016). It also makes sense as a suitable host for P450s, as it is a hydrophobic substrate‐utilizing yeast (Van Rensburg *et al*., 1997; Theron, 2012).

Also in agreement with our previous study, the *H. polymorpha* strain tested in this study was the only host for which no activity could be detected. A possible reason for the lack of activity is that the strain used during these studies is not as suitable for heterologous expression as the strains reported in the literature, as considerable variation in properties between different *H. polymorpha* strains has been reported (Böer *et al*., 2007). Expression of other reporter enzymes which were cloned into this strain also yielded poor results, although detectable activities were comparable to those obtained with transformants of *S. cerevisiae* strain W3031A(a) (Smit *et al*., 2012). In other studies, recombinant interleukin 6 production by *H. polymorpha* and *S. cerevisiae* resulted in truncated polypeptides compared to the correct, full‐length protein produced by *A. adeninivorans*; as well as heterologous expression of *Y. lipolytica* lipase by *H. polymorpha* was lower than *S*. *cerevisiae* and *A. adeninivorans* expression (Kumari *et al*., 2015). On the other hand, *H. polymorpha* was a better producer of a bacterial alcohol dehydrogenase than *A. adeninivorans* (Kasprzak *et al*., 2016). This re‐emphasizes the variable activities observed when different host strains are used for different heterologously expressed proteins.

The *K. marxianus* strain tested in this study exhibits high instability of integrants (Smit *et al*., 2012; Theron *et al*., 2014). The integration instability of this yeast, similar to other yeasts, has been attributed to a competition between the homologous recombination (HR) and non‐homologous end‐joining (NHEJ) mechanisms within the cell for the integration cassette, with deletion of either component of the Ku70p/Ku80p heterodimer in *K. marxianus* decreasing NHEJ efficiency and favouring HR efficiency (Kooistra *et al*., 2004; Abdel‐Banat *et al*., 2010). Integration instability could also be attributed to the ploidy of the strain used, as haploid strains tend to be more amenable to genetic manipulation than diploid strains (Lane and Morrissey, 2010). Therefore, it should be noted that while the results obtained in this study may be relevant in terms of the long‐term use of transformants of this strain, they are probably underestimations of the best possible activities achievable when using this yeast.

In some cases (particularly *A. adeninivorans* CYP53 only transformants and CYP53‐UmCPR combinations in *K. marxianus* and *Y. lipolytica*), there appeared to be fold differences in activities between transformants of the same host containing the same integrant. This could possibly indicate copy number differences between these transformants, but requires further confirmation. Copy number determination would require the identification of appropriate markers for quantitative PCR for each species, which have not yet been established (Terentiev *et al*., 2004), and was not directly part of the focus of this study. Nevertheless, with integration of similar cassette components into *A. adeninivorans* (i.e. a TEF promoter‐regulated hygromycin resistance marker, and rDNA elements as integration targets), 1–3 stably integrated copies were observed (Rösel and Kunze, 1998). In the case of *A. adeninivorans* transformants with CYP53B1 only, there appears to be a twofold difference between activities. Therefore, assuming there were copy number differences, these transformants would most probably contain one and two copies respectively. Since the potential single copy transformant in any case displayed far higher activities than any other yeast transformant in this study (eightfold higher than the next best *S. cerevisiae* transformant 2 in 24 h), copy number was ruled out as the main reason for superior activity of *A. adeninivorans*, since no tested transformant of any species could contain less than one copy of the integrant.

Activity differences between transformants could, however, also be due to different integration loci within the rDNA. Many of the repeats of rDNA subunits are silenced in order to maintain rDNA stability and protect the cell from damage associated with unnecessary overproduction of the transcripts of these units (Cioci *et al*., 2003; Ha *et al*., 2012). Reporter genes integrated within silenced rDNA would therefore also not be expressed, leading to lower activities than in other situations. Either way, for analytical purposes, we compared higher activity transformants between strains directly as ‘best case scenarios’ and lower activity transformants between strains directly as probable ‘single copy’ integrants.

The activities obtained with the *A. adeninivorans* transformants coexpressing the UmCPR and CYP53B1 were remarkably high if we consider that in the previous study where CYP53B1 was expressed in *Y. lipolytica* under the strong inducible POX2 promoter, the maximum specific activities obtained after optimization were only 0.08 U.g_DCW_^−1^ (Shiningavamwe *et al*., 2006), while the maximum specific activities reported here were as high as 0.19 U.g_DCW_^−1^ (Table S4). In 2016, Jeon *et al*. (2016) reported only 0.47 mM pHBA produced in 24 h reactions with a *S. cerevisiae* strain coexpressing the *F. oxysporum* CYP53 and CPR with the glucose dehydrogenase from *Bacillus megaterium* for cofactor regeneration. In the current study, the volumetric yields of the *A. adeninivorans* transformant coexpressing CYP53B1 and UmCPR after 24 h reactions were as high as 4.6 mM, approximately 10‐fold higher than that report (Table S4).

## Conclusion

This study confirmed our previous findings that *A. adeninivorans* is a promising host for recombinant P450 production as well as for whole‐cell biocatalysis. This opens the door to investigate the expression of other P450s using this host. It also demonstrated, for the first time to our knowledge, the improvement of P450 activity by coexpression with a non‐related CPR over the natural partner, thus highlighting the critical importance of CPR selection in P450‐based studies. As the roles and interactions of CPRs are currently receiving increasing attention, these findings are highly relevant in the quest to better understand these enzymes and their variable interactions with P450s.

## Experimental procedures

### Chemicals, strains and vectors

Chemicals and antibiotics were obtained from Merck (Johannesburg, South Africa). DNA modification enzymes were obtained from Thermo Scientific (Johannesburg, South Africa), New England Biolabs (Pretoria, South Africa), Lucigen (Johannesburg, South Africa) and Kapa Biosystems (Cape Town, South Africa). BioFlux Biospin gel extraction kits and Biospin plasmid DNA extraction kits for DNA/RNA extraction and purification were supplied by Separations Scientific (Johannesburg, South Africa).

*Escherichia coli* XL‐10 Gold (Stratagene) was used for cloning and plasmid propagation. Yeast strains used as hosts for heterologous expression are listed in Table 1 and were all obtained from the University of the Free State (UFS) culture collection.

**Table 1:** List of yeast strains used in this study

Yeast	Strain
*Saccharomyces cerevisiae*	W3031A(a)
*Yarrowia lipolytica*	CTY003^a^
*Kluyveromyces marxianus*	CBS 6556
*Hansenula polymorpha*	CBS 4732
*Arxula adeninivorans*	CBS 7350
*Rhodotorula minuta*	CBS 2177
*Ustilago maydis*	DSM 3121

a A derivative of *Y. lipolytica* E150 (Barth and Gaillardin, 1996) with leucine prototrophy restored (Theron, 2012; Theron *et al*., 2014).

The cDNA for CYP53B1 (NCBI accession number: D63703) was obtained from *Rhodotorula minuta* total RNA during a doctoral study in our group (Shiningavamwe, 2004) and was available in the plasmid JMP62‐53. cDNA for the cytochrome P450 reductase (CPR) from *Yarrowia lipolytica* (YlCPR; NCBI accession number: AB126598.1) was available from the same study in the plasmid JMP21‐CPR. These plasmids were used as templates for the PCR amplification of these genes together with the introduction of EcoRV (5′) and AvrII (3′) restriction sites. The resultant amplicons were cloned into the pSMART HC Kan subcloning vector (Lucigen), resulting in pSMART+CYP53B1 and pSMART+YlCPR before verification by sequencing.

Fresh total RNA was extracted from the same *Rhodotorula minuta* strain used previously to clone CYP53B1, to obtain the cDNA of the CPR (RmCPR; NCBI accession number: AB055119). Briefly, *R. minuta* was inoculated into 5 ml YPD broth (yeast extract 1% w/v, peptone 2% w/v, glucose 2% w/v) and incubated at 30°C for 48 h. The complete 5 ml culture was transferred to 100 ml yeast nitrogen base (YNB) broth supplemented with phenylalanine (final concentration of 1 g l^−1^). Total RNA was isolated using liquid nitrogen grinding and TriZol (Labuschagne and Albertyn, 2007) and reverse‐transcribed using the Roche Transcriptor High Fidelity cDNA Synthesis Kit, utilizing the oligo dT18 primer for first strand cDNA synthesis. The complete cDNA encoding the RmCPR ORF was PCR amplified and cloned into pSMART HC Kan (pSMART+RmCPR), followed by sequence verification.

Genomic DNA extracted from *Ustilago maydis* strain DSM3121 according to the method described by Labuschagne and Albertyn (2007) served as template to PCR‐amplify the CPR (UmCPR; NCBI accession number: XM_011389981.1). The resultant amplicon was subcloned into pSMART HC Kan (pSMART+UmCPR) and verified by sequencing.

### Construction of expression vectors

Standard molecular biology techniques were carried out as described by Sambrook and Russel (2001), and enzymes were applied according to the specifications of the manufacturers. Broad‐range yeast expression vectors pKM173 and pKM177 had been constructed in our group from components derived from yeast strains from the UFS culture collection (Smit *et al*., 2012; Theron *et al*., 2014). The two variations of the vector, pKM173 and pKM177, differ in the number of I‐SceI recognition sites present. pKM173 has only one site preceding the promoter YlTEFp, while pKM177 has two sites that flank the expression cassette on both sides (Smit *et al*., 2012; Theron *et al*., 2014). These vectors were previously used to introduce single or double CYP505A1 expression cassettes into *A. adeninivorans* (Theron *et al*., 2014).

In the current context, these vectors facilitated the coexpression of P450s and CPRs as follows: the CPRs were cloned into the multiple cloning site of pKM173 and CYP53B1 into pKM177 (as described below), followed by removal of the entire expression cassette from pKM177 and insertion into pKM173 using I‐SceI, resulting in a coexpression vector containing two distinct expression cassettes (Fig. S1).

CYP53B1 was removed from pSMART by digestion with EcoRV and AvrII, followed by treatment with T4 polymerase to generate blunt ends on both sides. The blunted product was ligated into pKM177 which had been digested with XhoI and AfeI, blunted by T4 polymerase treatment and dephosphorylated using Antarctic phosphatase. The ligation produced pKM177‐CYP53B1. pKM173 was digested with XhoI, followed by treatment with T4 polymerase. The resultant blunted product was then digested with AvrII. YlCPR was released from pSMART+YlCPR using EcoRV and AvrII, while RmCPR was released from pSMART+RmCPR using EcoRV and NheI. Both of the released products YlCPR and RmCPR were ligated into the prepared pKM173 vector, producing pKM173‐YlCPR and pKM173‐RmCPR respectively. UmCPR was removed from pSMART+UmCPR by digestion with SalI and AvrII, and ligated into pKM173 which was digested with XhoI and AvrII, producing pKM173‐UmCPR.

pKM177‐CYP53B1 was digested with I‐SceI, releasing the YlTEFp‐CYP53B1‐KmINUt expression cassette. The released expression cassette was ligated into each of the pKM173‐YlCPR, pKM173‐RmCPR and pKM173‐UmCPR vectors, all of which had been digested with I‐SceI. These ligations produced pKM173‐CYP53B1‐YlCPR, pKM173‐CYP53B1‐RmCPR and pKM173‐CYP53B1‐UmCPR; for the coexpression of CYP53B1 with each individual CPR.

### Transformation of yeasts

All yeast strains were transformed according to a modification of the method described by Lin‐Cereghino *et al*. (2005), except *K. marxianus* which was transformed according to a modification of the method of Chen *et al*. (1997). All yeasts were transformed with all of the vectors described in the previous section. Prior to transformation, the relevant vectors were digested with NotI to separate the yeast integration cassette from the bacterial moiety of the vector. After transformation, the cells were streaked on selective YPD plates (yeast extract 1% w/v, peptone 2% w/v, glucose 2% w/v, agar 2% w/v) supplemented with 400 mg/L hygromycin B and incubated until colonies appeared. Colonies were re‐streaked on selective YPD, inoculated into YPD broth and frozen with 15% v/v glycerol at −80°C. Genomic integration was confirmed by PCR using genomic DNA as template, extracted according to the method described by Labuschagne and Albertyn (2007).

### Biotransformations using recombinant yeast cells

Strains were revived from frozen stocks by streaking onto YPD selective plates. Cells from selective plates were used to inoculate YPD broth. Culture volumes were 5 ml per 25 ml test tube for initial screening, and 50 ml per 500 ml flask for further biotransformations. Inoculated cultures were incubated on a rotary shaker (130 rpm) at 28°C for 48 h, before addition of benzoic acid (BA), dissolved in dimethylsulfoxide (DMSO), as a substrate to final concentrations of 5 mM BA and 1% v/v DMSO; directly to the cultures. Cultures were incubated further as before and samples were taken over time.

Universal pH indicator strips (Merck) were used to monitor the pH values of the cultures.

Biomass determination was performed on 2 ml samples collected during cultivation in pre‐weighed microcentrifuge tubes. Cells were harvested at 13 000 × *g* for 5 min and washed with 0.9% w/v NaCl solution prior to wet cell weight (wcw) determination. The tubes were then baked at 100°C for 24 h, prior to dry cell weight (dcw) determination.

### Sample extraction and product analysis

Samples (500 μl) were taken at regular intervals and acidified using hydrochloric acid (5M) to below pH 3. Ethyl acetate (300 μl) containing myristic acid (0.5 mM), as an internal standard, was added to the samples. After thorough vortexing, samples were centrifuged for 10 minutes at 14 000 rpm. The upper organic layer was collected, before the process was repeated. For the assays carried out in amber vials using resting cells, the entire reaction mixture volume (2 ml) was extracted using proportionate amounts of ethyl acetate containing myristic acid (0.5 mM) as an internal standard. The collected organic extracts were pooled, and aliquots were concentrated using an Eppendorf Concentrator Plus, prior to further analysis.

Analysis was done using thin‐layer chromatography (TLC) and/or gas chromatography (GC). For TLC analysis, aliquots (5–10 μl) of organic extracts from samples and standards were spotted on Alugram^®^ silica gel F_254_ TLC plates (Merck), and the plates were developed using a mobile phase containing di‐*n*‐butyl ether: formic acid: distilled water (90:7:3 v/v/v). The plates were dried and viewed under UV light. Aromatic compounds appeared as dark spots due to UV absorption. TLC plates were analysed using a Bio‐Rad Gel Doc EQ system with Quantity One^®^ version 4.5 software.

Organic acids were methylated prior to GC analysis using equal volumes of a trimethylsulfonium hydroxide (TMSH) preparation (Obiero, 2006). GC analyses were performed on samples (1 μl) using a Hewlett‐Packard 5890 series II gas chromatograph equipped with a 30 m (length) × 0.25 mm (inner diameter) × 0.25 μm (film thickness) SGE BPX20 column, with carrier gas, H_2_, at 5 ml/min, split ratio of 1:40, inlet temperature at 250°C, initial temperature at 120°C for 15 min, increased at 5°C/min to 250°C, which was maintained for 5 min. Flame ionization detector (FID) temperature was at 300°C.

## Conflict of interest

All authors declare that they have no conflict of interest.

## Availability of data and materials

The data sets generated and analysed during the current study are available from the corresponding author on reasonable request.

## Authors’ contributions

CWT and ML carried out vector construction and cloning of genes. CWT did experimental design and performed all yeast transformations, biotransformation experiments and analysis of data. CWT also drafted the initial manuscript. JA assisted with vector design and selection of yeast strains. MS conceived the study and aided in the experimental design and data analysis. MS edited the final manuscript. All authors read and approved of the final manuscript.

## Supporting information


**Fig. S1.** Schematic representation of the construction of coexpression vectors.

**Table S1.** Oligonucleotides used for amplification of reporter genes.

**Table S2.** Typical biomass concentrations of transformants of the different yeast strains just prior to substrate addition and pH of cultures at this time and when 24 h biotransformation samples were taken.

**Table S3.** Comparison of specific activities of *A. adeninivorans* transformants calculated based on the 24 h sample and those determined as the gradients of progress curves.

**Table S4.** Comparison of CYP53B1 activities obtained using transformants from different strains with and without coexpression of UmCPR.

**Table S5.** Differential effects of co‐expressed CPRs on recombinant CYP53B1 activities in *A. adeninivorans* transformants.
