# Supplementary material for: Heterologous coexpression of the benzoate‐para‐hydroxylase CYP53B1 with different cytochrome P450 reductases in various yeasts
Source: Microb Biotechnol. 2018 Oct 19;12(6):1126–38. doi: 10.1111/1751-7915.13321 (PMC6801163; doi:10.1111/1751-7915.13321)
Supplement: Supplementary file 1 — Fig. S1. Schematic representation of the construction of coexpression vectors. [file MBT2-12-1126-s001.pdf]

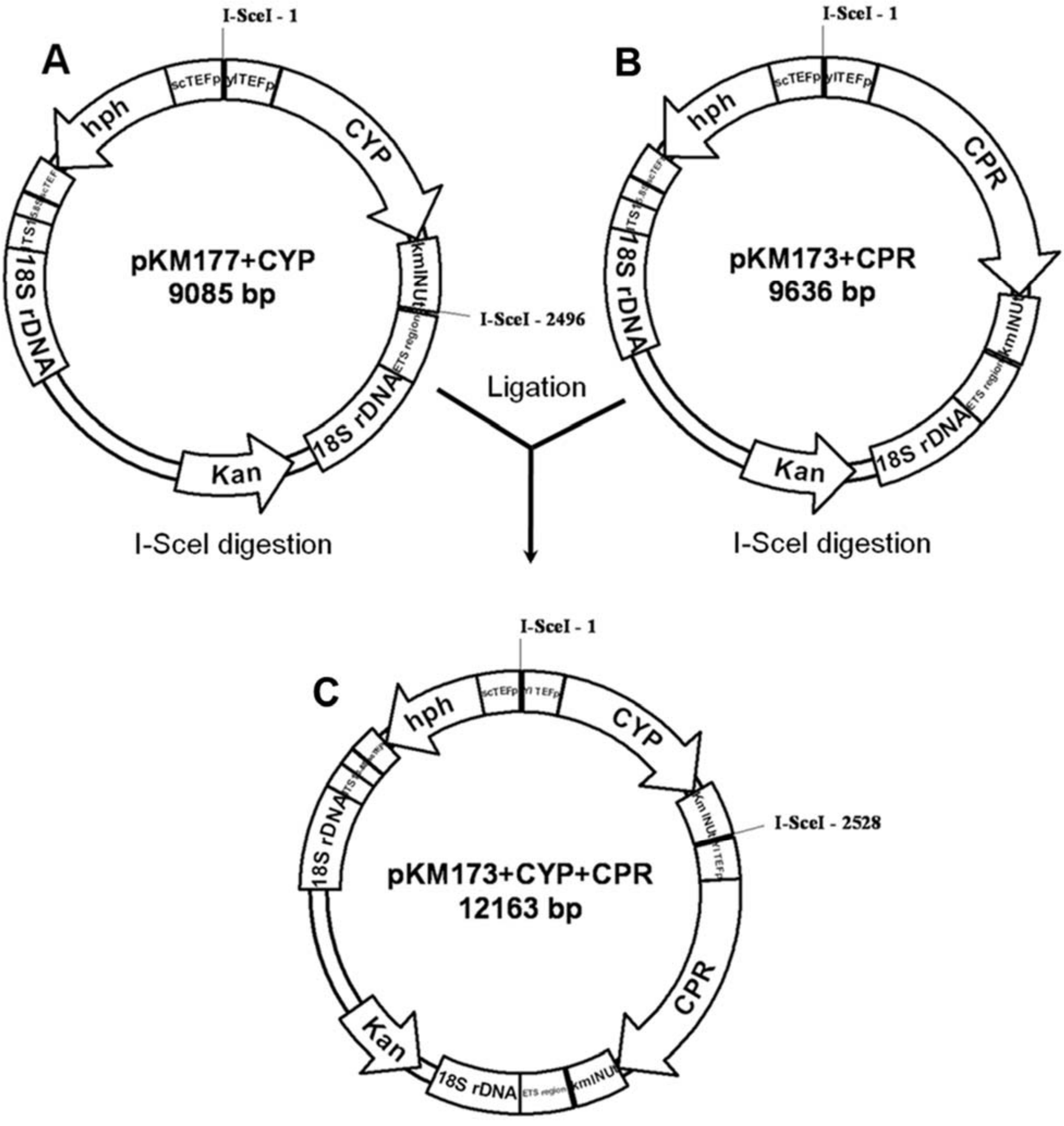

Figure S1: Schematic representation of the construction of coexpression vectors. pKM177 carrying CYP53B1 (A) and pKM173 carrying a CPR (B), were digested with I-*Sce*I. The CYP53B1-containing expression cassette released from pKM177 was ligated into the opened pKM173-CPR backbone, to yield the coexpression vector (C).
