# Supplementary material for: Heterologous coexpression of the benzoate‐para‐hydroxylase CYP53B1 with different cytochrome P450 reductases in various yeasts
Source: Microb Biotechnol. 2018 Oct 19;12(6):1126–38. doi: 10.1111/1751-7915.13321 (PMC6801163; doi:10.1111/1751-7915.13321)
Supplement: Supplementary file 2 — Table S1. Oligonucleotides used for amplification of reporter genes. [file MBT2-12-1126-s002.pdf]

Table S1. Oligonucleotides used for amplification of reporter genes.

**Primer name**	**Sequence in 5' to 3' orientation**	**Restriction sites introduced**	**IDT Tm (ºC)**
CYP53B1-1F	GATATCATGGGCATAGTCCAAGAAGCCG	*Eco*RV	60.6
CYP53B1-1R	CCTAGGCTAGGCATCAATGGATCTGCG	*Avr*II	62.4
ylCPR-1F	GATATCATGGCTCTACTCGACTCTCTCGAC	*Eco*RV	60.3
ylCPR-1R	CCTAGGCTACCACACATCTTCCTGGTAG	*Avr*II	61.2
U_maydis CPR 1F	GTCGACATGGCTTCGCAACTCGACTTGTTCGTTCTC	*Sal*I	66.3
U_maydis CPR-1R	CCTAGGTTAGGACCATACATCGAGAAGCAGCCTCG	*Avr*II	65.4
R_minuta CPR-1F	GATATCATGGAGTTCTCAACTAGCGACTACGTCCTCC	*Eco*RV	63.4
R_minuta CPR-1R	GCTAGCCTAACTCCAGACATCGAGGAGGAGACG	*Nhe*I	65.6
