# Supplementary material for: Heterologous coexpression of the benzoate‐para‐hydroxylase CYP53B1 with different cytochrome P450 reductases in various yeasts
Source: Microb Biotechnol. 2018 Oct 19;12(6):1126–38. doi: 10.1111/1751-7915.13321 (PMC6801163; doi:10.1111/1751-7915.13321)
Supplement: Supplementary file 3 — Table S2. Typical biomass concentrations of transformants of the different yeast strains just prior to substrate addition and pH of cultures at this time and when 24 h biotransformation samples were taken. [file MBT2-12-1126-s003.pdf]

Table S2: Typical biomass concentrations of transformants of the different yeast strains just prior to substrate addition and pH of cultures at this time and when 24 h biotransformation samples were taken.

Yeast host	Biomass after 48 h growth (just prior to BA addition) (g L_{DCW}^{-1})[a]	pH after 48 h growth (just prior to BA addition)	pH after 72 h growth (when 24 h biotransformation samples were taken)
K. marxianus	1 - 11	6	5.5
S. cerevisiae	4 - 6	5.5	5
Y. lipolytica	13 - 16	7	7
A. adeninivorans	17 – 18	8	8

[a]Ranges for four cultures (two of each of two transformants) grown in YPD medium.
