# Supplementary material for: Heterologous coexpression of the benzoate‐para‐hydroxylase CYP53B1 with different cytochrome P450 reductases in various yeasts
Source: Microb Biotechnol. 2018 Oct 19;12(6):1126–38. doi: 10.1111/1751-7915.13321 (PMC6801163; doi:10.1111/1751-7915.13321)
Supplement: Supplementary file 4 — Table S3. Comparison of specific activities of A. adeninivorans transformants calculated based on the 24 h sample and those determined as the gradients of progress curves. [file MBT2-12-1126-s004.pdf]

Table S3: Comparison of specific activities of *A. adeninivorans* transformants calculated based on the 24 h sample and those determined as the gradients of progress curves.

	**Specific activity (µmol. h^{-1}. g_{DCW}^{-1})**	
***A. adeninivorans* transformant (culture)**	**Calculated using 24 h sample**	**Determined from the gradient of a progress curve**
T4 (A)	8.85	8.97
T4 (B)	9.21	9.21
T5 (A)	11.01	11.05
T5 (B)	11.51	11.75
