# Supplementary material for: Heterologous coexpression of the benzoate‐para‐hydroxylase CYP53B1 with different cytochrome P450 reductases in various yeasts
Source: Microb Biotechnol. 2018 Oct 19;12(6):1126–38. doi: 10.1111/1751-7915.13321 (PMC6801163; doi:10.1111/1751-7915.13321)
Supplement: Supplementary file 5 — Table S4. Comparison of CYP53B1 activities obtained using transformants from different strains with and without coexpression of UmCPR. [file MBT2-12-1126-s005.pdf]

Table S4: Comparison of CYP53B1 activities obtained using transformants from different strains with and without coexpression of UmCPR.

Heterologous genes	Transformant	Average activity [a]	
		Volumetric yield after 24 h (mM)	Specific activity (µmol.h^{-1} g$_{DCW}$$^{-1}$)
**CYP53B1 only**	*K. marxianus* T2	1.97x10^{-2} ± 4.5 x10^{-3}	7.53x10^{-2} ± 6.3x10^{-3}
	K. marxianus T3	2.42x10^{-2} ± 9.7x10^{-3}	9.37x10^{-2} ± 1.5x10^{-2}
	S. cerevisiae T2	6.06x10^{-2} ± 4.1x10^{-5}	0.51 ± 4.8x10^{-2}
	S. cerevisiae T3	5.59x10^{-2} ± 9.6x10^{-3}	0.47 ± 8.2x10^{-2}
	Y. lipolytica T1	1.49x10^{-2} ± 9.6x10^{-3}	4.31x10^{-2} ± 4.4x10^{-3}
	Y. lipolytica T4	2.04x10^{-2} ± 1.5x10^{-3}	6.09x10^{-2} ± 6.7 x10^{-3}
	A. adeninivorans T1	0.50 ± 2.1x10^{-2}	1.18 ± 3.8x10^{-2}
	A. adeninivorans T2	0.91 ± 7.6x10^{-2}	2.17 ± 9.5x10^{-2}
**CYP53B1 + UmCPR**	*K. marxianus* T4	1.38x10^{-2} ± 5.2x10^{-4}	0.06 ± 7.8x10^{-4}
	K. marxianus T5	3.17x10^{-2} ± 3.3x10^{-}	0.13 ± 1.1x10^{-2}
	S. cerevisiae T3	0.11 ± 1.5x10^{-2}	0.79 ± 8.7x10^{-2}
	S. cerevisiae T4	0.12 ± 9x10^{-2}	0.76 ±0.5
	Y. lipolytica T3	0.31 ± 1.3x10^{-2}	0.70 ± 5.1x10^{-2}
	Y. lipolytica T5	0.12 ± 1.2x10^{-2}	0.25 ± 2x10^{-2}
	A. adeninivorans T4	3.72 ± 5x10^{-2}	9.03 ± 0.3
	A. adeninivorans T5	4.57 ± 9.5x10^{-2}	11.3 ± 0.35

[a] Average CYP53B1 activities of duplicate cultures were determined.
