# Supplementary material for: Heterologous coexpression of the benzoate‐para‐hydroxylase CYP53B1 with different cytochrome P450 reductases in various yeasts
Source: Microb Biotechnol. 2018 Oct 19;12(6):1126–38. doi: 10.1111/1751-7915.13321 (PMC6801163; doi:10.1111/1751-7915.13321)
Supplement: Supplementary file 6 — Table S5. Differential effects of co‐expressed CPRs on recombinant CYP53B1 activities in A. adeninivorans transformants. [file MBT2-12-1126-s006.pdf]

Table S5: Differential effects of co-expressed CPRs on recombinant CYP53B1 activities in *A. adeninivorans* transformants.

**CPR activity**	**Transformant**	**Average activity [a]**	
		**Volumetric yield (mM)**	**Specific activity (μmol.h^{-1} g$_{DCW}$$^{-1}$)**
Native only	T2	0.54 ± 7.8x10^{-2}	1.31 ± 0.2
	T5	0.37 ± 8.8x10^{-3}	0.96 ± 2.7x10^{-2}
Native + YlCPR	T4	0.23 ± 4.4x10^{-3}	0.59 ± 4x10^{-3}
	T5	0.45 ± 1.3x10^{-2}	1.13 ± 4.1x10^{-2}
Native + RmCPR	T3	1.75 ± 0.1	4.37 ± 0.3
	T4	1.99 ± 0.2	4.93 ± 0.5
Native + UmCPR	T4	2.70 ± 0.1	6.74 ± 0.5
	T5	3.35 ± 0.1	8.11 ± 0.2

[a] Average CYP53B1 activity of duplicate cultures.
